# Quantitative study of spatial and temporal variation in retinal capillary network perfusion in rat eye by in vivo confocal imaging

**DOI:** 10.1038/s41598-023-44480-1

**Published:** 2023-11-02

**Authors:** Paula Kun Yu, Andrew Mehnert, Jayden Brendan Dickson, Hassanain Qambari, Chandrakumar Balaratnasingam, Stephen Cringle, Dean Darcey, Dao-Yi Yu

**Affiliations:** 1 Centre for Ophthalmology and Visual Science, The University of Western Australia, Perth, Australia; 2 Lions Eye Institute, 2 Verdun Street, Nedlands, WA Australia; 3 Department of Ophthalmology, Sir Charles Gairdner Hospital, Nedlands, WA Australia

**Keywords:** Biological techniques, Physiology, Medical research

## Abstract

Microvascular dysfunction is the underlying pathological process in many systemic diseases. However, investigation into its pathogenesis is impeded by the accessibility and complexity of the microvasculature within different organs, particularly for the central nervous system. The retina as an extension of the cerebrum provides a glimpse into the brain through which the microvasculature can be observed. Two major questions remain unanswered: How do the microvessels regulate spatial and temporal delivery to satisfy the varying cellular demands, and how can we quantify blood perfusion in the 3D capillary network? Here, quantitative measurements of red blood cell (RBC) speed in each vessel in the field were made in the in vivo rat retinal capillary network using an ultrafast confocal technique with fluorescently labelled RBCs. Retinal RBC speed and number were found to vary remarkably between microvessels ranging from 215 to 6641 microns per second with significant variations spatially and temporally. Overall, the RBC speed was significantly faster in the microvessels in the superficial retina than in the deep retina (estimated marginal means of 2405 ± 238.2 µm/s, 1641 ± 173.0 µm/s respectively). These observations point to a highly dynamic nature of microvasculature that is specific to its immediate cellular environment and is constantly changing.

## Introduction

The microcirculation comprises a capillary network, the supplying arterioles (up to ~ 100 µm internal diameter) and the corresponding draining venules and veins (somewhat larger than the corresponding arterioles), embedded within specific organs for the exchange of nutrients and waste^1^ at the cellular level. Microcirculation is physically present near almost every living cell and has a critical role in modulating vascular tone to match local oxygen demand. Malfunction of the microcirculation or microcirculation dysfunction is a major contributor to a myriad of morbidities and mortalities^2, 3^ such as ischemic cardiomyopathy, diabetes mellitus, cerebral vasospasm and dementia to name a few. Microvascular dysfunction includes vessel destruction, abnormal vasoreactivity, in situ thrombosis and fibrosis. In the eye, microcirculation dysfunction may eventually manifest as retinal occlusive diseases, diabetic retinopathy^4, 5^, and glaucoma^6^.

The retinal microcirculation has a unique topology to nourish and support one of the most metabolically active tissue in the body. The retina is highly structured and has a compact layering of neuronal, synaptic, and vascular elements with layer specific and varying demands. Ailments such as visual field progression in glaucoma^7^, pathological myopia^8^, and diabetic retinopathy^9^ have noted layer specific changes in vessel density and altered perfusion as measured by existing clinical OCTA devices and experimental techniques utilizing microsphere, laser doppler measurement and horseradish peroxidase staining. Whilst many techniques^10–12^ are available to study macrovascular^13^ and retinal vessel changes^14–16^, non-invasive quantitative microvascular investigation at the capillary level has proven challenging. Flow speed measurements obtained using laser Doppler velocimetry, scanning laser Doppler flowmetry, and optical coherent tomography angiography (OCTA) are largely based on the major retinal arterioles and veins located superficially on the retina. This limits the ability to obtain layer specific quantitative information especially from the deep capillary plexus. Given the unique high metabolic demand microenvironment and the function specific layering of the neurons, it is important to understand how microvascular dysfunction may develop and progress in the layer specific retina to formulate suitable therapeutic strategies.

In the present work, we applied a confocal technique in combination with fluorescently labelled red blood cells (fRBCs) to image red blood cell movement in a live rat retinal capillary network. Red blood cell movement in different retinal capillary networks could then be quantified and further analysed spatially and temporally, and specific to the vascular layer within the rat retina. The red blood cell speeds measured are comparable to reported values obtained from single vessel studies. Our results show that temporal and spatial variation in fRBC speed is normally present in the absence of additional metabolic challenge to the air breathing rat. The variation observed likely reflects the natural retinal microvascular flow which can be altered in metabolic and pathologic challenges.

## Results

### Rat physiologic indices

Table 1 lists the mean and SD body weight, pH, pO_2_, pCO_2_, blood pressure, heart rate and blood glucose of the 8 rats used in this study. The pH and pCO_2_ are within normal physiological range. The blood glucose of the rats during the experiment was noted to be elevated, averaging 28 ± 3.6 mM, when these rats had not been induced for diabetes. Further investigation identified that medetomidine used in this study to be the likely cause as similar elevation in blood glucose was reported by Connell et al. in their search of a suitable anaesthetic for study of rat ERGs^17^.

**Table 1:** Physiological indices (mean and SD) of the 8 rats.

	Body Weight (g)	pH	pO_2_ (mmHg)	pCO_2_ (mmHg)	Blood Pressure (mmHg)	Heart Rate (BPM)	Blood Glucose (mmol/L)
Mean	530	7.4	75.6	36.6	98	241	27.6
SD	47.4	0.03	15.27	5.73	33.4	74.2	3.57

### In vivo imaging

The average volume of blood drawn from the femoral artery line for red blood cell labeling was 582.5 ± 137.81 µl (n = 8). The red blood cells were segregated from plasma using a centrifuge and resuspended in a buffered solution of lipophilic dye (DiD; Invitrogen D-7757) at 31.25 µg/ml for 5 min incubation at 37 degrees Celsius for labelling^18^. The labelled red blood cells, fRBC, were spun down and washed from unbound dye before they were injected back into the rat circulation through the jugular vein line.

**Figure 1:**
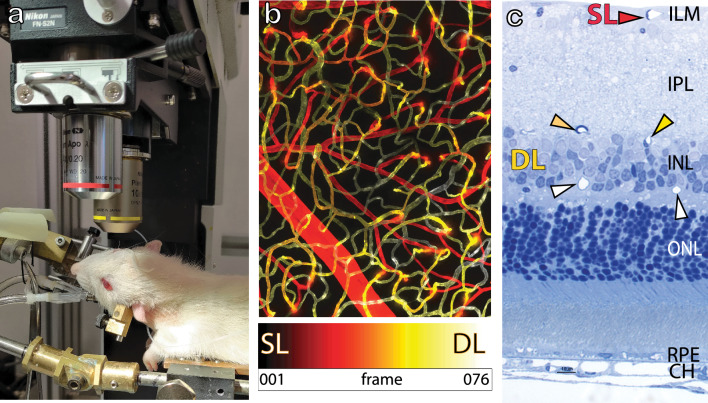
Experiment set-up. (**a**) Image shows the right eye of a rat undergoing in vivo imaging using the Nikon A1 system with a × 10 Plan Apo objective lens. The rat is anaesthetized and the trachea intubated for artificial ventilation and its right eye aligned with the objective lens. (**b**) shows a projected stack of confocal images from the superficial (SL) and deep (DL) vascular plexus within the rat retina at approximately 2 disc distance region. The rat retinal vasculature was perfuse labeled using FITC-Lectins. The image stack was depth color-coded from frame 1–76 (SL to DL) such that the most superficial vessels are in red and the deepest vessels in white as indicated in the color bar below. (**c**) is a semi-thin epoxy transverse section of a rat retina, stained with toluidine blue. Arrow heads point to retinal vessels seated within the retinal neuronal layers. The SL is embedded in the most anterior portion of the retina close to the inner limiting membrane (ILM) as indicated by the red arrow head. Two closely apposed layers are seated on the anterior and poster borders of the inner nuclear layer (INL) and together are considered as DL for in vivo imaging. ONL, IPL, RPE and CH refers to the outer nuclear layer, inner plexiform layer, retinal pigmented epithelial cells and the choroid.

**Figure 2:**
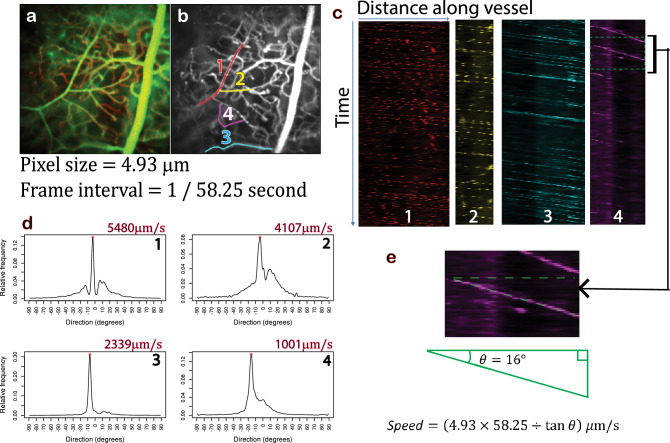
Kymograph analysis method. (**a**) Temporal projection over 292 frames of an in vivo time series (corresponding to the first 5 s of Movie S1). The pixel size is 4.93 µm and the frame rate is 58.25 fps. (**b**) Pixel-wise standard deviation of all frames in the Cy5 channel (red channel showing fRBCs). Centreline paths have been manually traced for four vessel segments from upstream to downstream according to fRBC flow direction. (**c**) Kymographs generated for each of the four vessel segments (colorised here for easy cross reference with (**b**)). Each kymograph is a 2D image with the first row containing the Cy5 channel intensities (ordered from left to right) along the centreline path in the direction of flow for the first frame, the second row containing those from the second frame and so on. The high intensity streaks within each kymograph reflect fRBC movement through time. Moving fRBCs appear as diagonal streaks. (**d**) Histograms indicating the amount of structure present at each angle within each kymograph. The peak in each histogram, marked with a red circle, corresponds to the dominant streak direction and is used to calculate fRBC speed for the corresponding vessel segment (speed is shown at the top of each histogram). (**e**) Magnified portion of kymograph 4 in C demonstrating the calculation of flow speed using the dominant streak direction from histogram 4 in (**d**). This corresponds to the angle of declination of the streak (see the the overlaid dotted green triangle). The fRBC speed is then given by 4.93 × 58.25 ÷ tan (16°) ≈ 1001 μm/s.

Figures 1 and 2 show the experimental set-up and illustrate the spatiotemporal kymograph^19–22^ analysis method used to estimate fRBC velocity respectively. The FITC-Dextran filled retinal microvascular network was clearly visualised under epifluorescence and through in vivo imaging. Data^23^ were captured from the superficial (SL) and deep (DL) retinal vessels as defined in Fig. 1b,c. The fRBCs can be clearly seen flowing through the retinal microvascular network in the acquired time series (e.g., Fig. 2a, Supporting information file: Movie S1). Note that the DL includes both the intermediate and deep capillary plexus as indicated in Fig. 1c.

### Spatial and temporal variation in fRBC speeds

**Figure 3:**
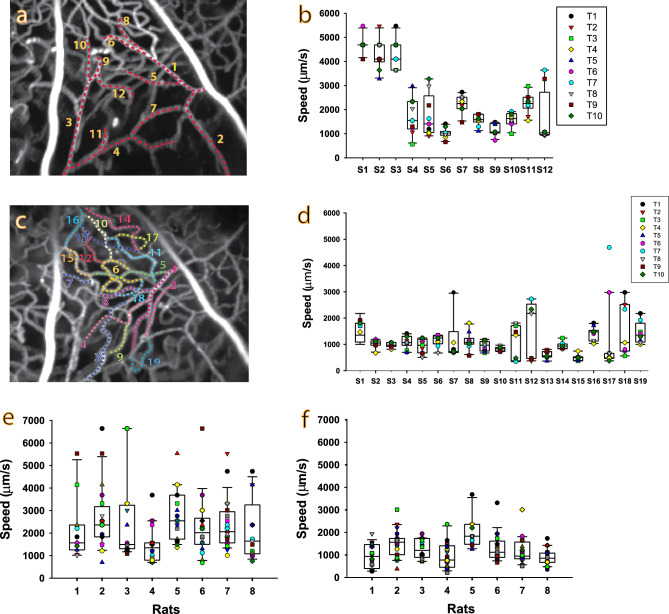
Spatio-temporal variation in fRBC flow speed—representative data and overall data. (**a**) and (**c**) show the standard deviation temporal projections of the fRBC signal for 30 s video sequences of the superficial (SL) and deep layers (DL) respectively. The overlaid color dotted lines show the vessel segments belonging to the SL or DL selected according to histology for measurement. (**b**) and (**d**) are plots of the kymograph-derived fRBC speeds computed for consecutive 3 s intervals (T1–T10) for each of the vessel segments (S1–S19) highlighted in (**a**) and (**c**) respectively. A large range of fRBC speeds is present in the vessel segments from both retinal microvascular layers as indicated by the variation in box plot medians between the vessel segments, especially in the SL vessels in (**b**). Less variation in speed was observed in the DL vessel segments as indicated in (**d**). The overall range of speeds found in the SL, (**b**), are higher than those found in DL, (**d**). Temporal variation in speed is evidenced from the spread of the speeds for each vessel segment (S) displayed as per time interval (T1 to T10). (**e**) and (**f**) show box plots of fRBC speed for the SL and DL vessels segments respectively for each rat. Each symbol overlaid on the box plots represents an averaged fRBC speed of a single vessel segment of that rat. Like the representative case shown in (**a**)–(**d**), the absolute and temporal variation in speeds detected in the SL, (**e**), are higher than those observed in the DL, **(f)**, for all 8 rats studied.

The speed of fRBC movement was determined in retinal microvessels located at two-disc distance away from the optic disc. A large range of fRBC speeds were found between vessels in the SL and DL vascular layers. Speed variation over time was found in almost all vessel segments studied. Such spatial and temporal variation in flow speeds was present in the SL and DL of all the rats examined. Figure 3a–d show a representative set of data from the SL and DL of one of the rats in this study. Figure 3a,c show the pixel-wise standard deviation of all the aligned Cy5 frames from 30 s video sequences of the SL and DL respectively. Trajectories through selected vessels are shown as dashed lines. Figure 3b,d show side-by-side box plots of the corresponding fRBC speeds computed for consecutive 3 s intervals for each of the vessels. There is clear temporal and spatial variation in speeds. Vessel segments were classified as arterioles, capillaries or venules based on the flow direction of red blood cells on the in vivo time series, and the presence or absence of vascular smooth muscle cells on the histology preparation of that vessel segment. Each vessel segment, designated “S”, was assigned a number for identification. Notably, in the SL the speeds in the arterioles (S1, S2 and S3) are higher than those in the capillaries (S6 and S9). Similarly, in the DL the speed in the arteriole (S1) is higher than those in the capillaries (S4, S5 and S8).

Figure 3e,f show fRBC speeds in all selected vessels, computed from 30 s time series, in the SL and DL for each of the 8 rats. The SL fRBC speeds range from 683 to 6641 µm/s (total of 141 vessels analysed). The DL fRBC speeds range from 215 to 3684 µm/s (total of 135 vessels). A linear mixed effects model (LMM) was fitted to the speed data. The mean speed in the SL was 2405 ± 238.2 µm/s (estimated marginal mean ± standard error), significantly faster than the mean speed in the DL which was 1641 ± 173.0 µm/s (1-tail test, p < 0.0001; see Materials & Methods for details in relation to the fitted linear mixed effects model and post-hoc comparison test).

### Spatial and temporal variation in haematocrit

**Figure 4:**
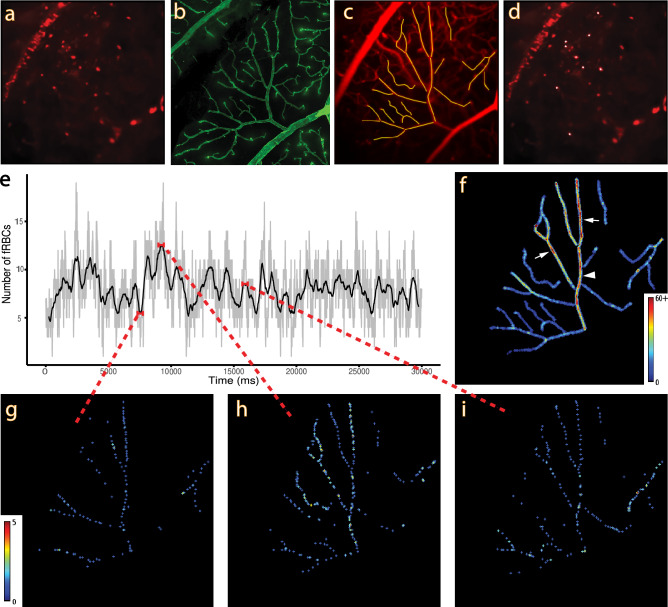
Spatio-temporal variation in capillary plexus fRBC count—representative data. (**a**) Single frame (~ 17 ms) from a 30 s fRBC time recording. Fluorescent labelled red blood cells may be observed as red spots. (**b**) Histology corresponding to the field of view in (**a**). (**c**) Pixel-wise standard deviation over all frames, superimposed with the centrelines of vessels selected using (**b**) as a guide. (**d**) is the image in (**a**) superimposed with white crosses showing the fRBC candidates selected by the spot detection algorithm that coincide with the selected vessels. (**e**) is a plot of fRBC count (light grey) frame-by-frame for all selected vessels, superimposed with a ~ 0.5 s moving average (black). Variation in the number of fRBCs can be observed throughout the time series. (**f**) is a fRBC count map created by assigning the value 1 to the pixels of each white cross (detected fRBC) in each frame, and then computing the pixel-wise sum over all frames. The fRBC count map has been color-coded to visualize the variation in cumulative counts in different vessel segments. Variation in cumulative fRBC counts can be observed at different locations along the same vessel segments. The highest cumulative fRBC count may be seen in the main trunk of the supplying arteriole (white arrowhead) and two of the side branches (white arrows). (**g**), (**h**) and (**i**) show count maps for a 5 s interval as marked in (**e**). These images exemplify the time and space dependent differential distribution of fRBCs. The color bar in (**g**) applies also to (**h**) and (**i**).

The fRBC count was used as a proxy measure of haematocrit, which is an important hemorheological property of the microvasculature. Based on Probst et al.^24^ reference values of blood volume (6.86 ± 0.53 ml/100 g) in male SD rats at 12 weeks of age, the average volume of labelled fRBCs is 1.72 ± 0.30%. The measured fRBC counts over time demonstrated clear temporal and spatial variation in both SL and DL in all rats examined. Figure 4 shows a representative set of data from the SL of a rat in this study. Figure 4a–d demonstrate the steps involved in the vessel segment selection based on correspondence with the histology image (Fig. 4b), and a representative image showing the fRBC candidates identified by the spot detection algorithm (Fig. 4d) for inclusion in the cell count within a single frame (Fig. 4a). Figure 4e shows a plot of the total fRBC number in the selected vessels in each frame over 30 s. The light grey curve corresponds to the raw counts and the black curve is a ~ 0.5 s moving average (smoothed version of the grey curve). The plot demonstrates temporal variation in fRBC counts. Figure 4f shows the fRBC count map (cumulative fRBC count at each pixel location) over 30 s, demonstrating spatial variation in fRBC counts. The main trunk of the supplying arteriole and two of the side branches show a higher cumulative fRBC count (more red dots, Fig. 4f) than the other vessel segments, likely indicating a higher flow volume, hence higher occurrence of fRBC, through these three vessels compared to the other vessel segments. Figure 4g–i show count maps for a 5 s interval centred at each of the timepoints as indicated. The maps demonstrate clear temporal and spatial variation in fRBC counts.

### Vessel diameter versus fRBC speed

To investigate whether the range of fRBC speed is related to the vessel diameter of each vessel segment and the vessel type, vessel diameters measurements were obtained from vascular histology and in vivo images for the same vessel segments. Some of the in vivo vessels could not be measured accurately due to effects of aberration within a relatively thick in vivo confocal image. Some of the micro vessels failed to be labelled by perfusion with Lectin. These vessels were excluded from the investigation.

The mean in vivo diameters are from around 2–2.5 times larger than the corresponding mean histology diameters and are much more variable. The inability of the in vivo technique to measure vessel diameter is likely due to two reasons. Firstly, the in vivo images have a resolution of 1.23 µm per pixel compared to between 0.31 and 0.62 µm per pixel for the histology images. This much poorer resolution likely limits accurate detection / delineation of the vessel walls. Secondly, denoising and interpolation as part of the frame alignment step, yielded expanded vessel extent in the in vivo frames used for measurement.

**Figure 5:**
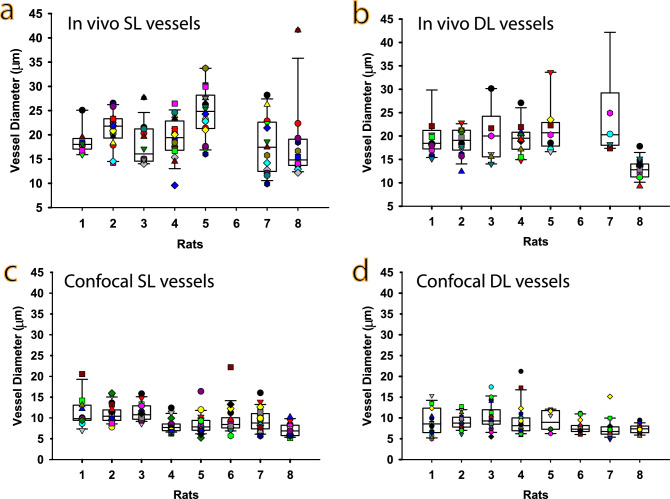
Diameter measurements from in vivo and confocal images. (**a**) & (**b**) are box plots of vessel diameter for the SL and DL computed from the higher resolution in vivo images. Data was not collected for rat 6. (**c**) & (**d**) are box plots of vessel diameter of the same vessels measured from histology images. The colored symbols overlaid on the box plots are diameter measurements of each vessel.

Figure 5a–d show vessel diameter, obtained from both the in vivo images (5a and b) and histology images (5c and d), for the SL (5a and c) and DL (5b and d) for each rat. A LMM was fitted to this data (see Materials & Methods for details). Table 2 shows the estimated marginal mean ± standard error (SE) diameter for each vessel type and layer. Notably, the mean diameter of capillaries in SL and in DL, measured using histology, are not significantly different (2-tail test, p = 0.141; see Materials & Methods for details in relation to the fitted LMM and post-hoc comparison test). Next, a LMM was fitted to the combined speed and diameter data to investigate whether vessel diameters measured in vivo or from histology were predictive of fRBC speed measurements (see Materials & Methods for details). The most parsimonious model does not include in vivo diameter as a predictor. The correlation between fRBC speed and histology diameter measurements was computed separately for both layers, the DL and the SL using repeated measures correlation (accounts for the non-independence of the multiple paired measurements per rat). The correlation coefficients were: ${r}_{\mathrm{both}}=0.59$, ${r}_{\mathrm{DL}}=0.50$, and ${r}_{\mathrm{SL}}=0.69$ respectively. The correlation coefficients are all positive showing moderate to strong correlation between (histology) vessel diameter and fRBC speed. The strongest correlation is in the superficial layer and the weakest in the deep layer.

**Table 2:** fRBC flow speed and vessel diameter measurements from the confocal images and in vivo images for the superficial layer (SL) and deep layer (DL).

Vessel segment type	fRBC speed (mean ± SE) in µm/s	Confocal diameter (mean ± SE) in µm	In vivo image diameter (mean ± SE) in µm
SL vessel segments			
A2	6728 ± 1709.4	14.3 ± 1.62	35.5 ± 5.30
A3	3043 ± 392.7	10.6 ± 0.54	23.9 ± 1.57
A4	2582 ± 274.4	9.8 ± 0.41	21.7 ± 1.19
Capillaries	1509 ± 137.0	8.2 ± 0.27	17.2 ± 0.73
Capillaries direct to venule	926 ± 237.1	7.3 ± 0.76	15.0 ± 1.96
V2	2631 ± 392.9	13.8 ± 0.96	33.9 ± 3.10
DL vessel segments			
Capillaries	1030 ± 89.4	7.9 ± 0.24	16.6 ± 0.67
V3	1634 ± 251.3	11.2 ± 0.72	25.8 ± 2.16
V2	1795 ± 262.6	13.3 ± 0.88	32.2 ± 2.83

Reported speeds are estimated marginal means and their standard errors (SEs) from the fitted linear mixed models.

## Discussion

Heterogenic capillary perfusion has previously been demonstrated in the heart using tracer techniques^25, 26^. Spatial and temporal heterogeneities of capillary hemodynamics in the brain have been postulated to be the result of heterogenous movement of microvascular haematocrit and heterogenous increase of temporal RBC fluctuation^27^. More recently, analysis of optical coherence tomography angiography images in human macula have also identified the presence of spatial and temporal variation in retinal microvessels^28^. The findings from the current study provide solid evidence of such variations through real confocal time-series images and quantitative measurements obtained from the live rat retina.

The current study captured live recordings of retinal blood flow from confocal imaging of fluorescently labelled red blood cells (fRBCs) and plasma. Fields of view measuring 1.59 square millimetres were captured using a × 10 objective lens with a frame rate sufficient to measure fRBC velocities in retinal microvessels. Analysis of fRBC speeds identified significant time and space dependent variation in each of the vessel segments examined. A significant difference in flow speed was identified between the two vascular plexuses with speed in SL (estimated marginal mean of 2405 ± 238.2 µm/s) almost 1.5 times that of the DL (1641 ± 173.0 µm/s) when breathing room air. The range of fRBC speeds is comparable to the published data obtained from particle image velocimetry analysis of adaptive optics images of human retinal capillaries^29^. The 50% higher speed for RBCs in the SL compared to DL also concurs with published data^18^ from single vessel studies where RBC flux (RBC count per second) was also found to be some 50% higher in the SL compared to the intermediate and deep vascular layers. The range of measured speeds is likely influenced by the vascular network topology, vessel order and type, and to a smaller extent the vessel diameter.

In addition to heterogeneity of blood flow, significant spatial and temporal heterogeneity of haematocrit has been demonstrated. Within the microvasculature, the mean red cell speed would be higher than the mean speed of the suspension, because of the Fåhraeus effect, leading to a reduction in haematocrit and increase in plasma gap. The viscosity of blood would also change as a result. Although Fåhraeus^30^ realised the importance of haematocrit reduction in his tube study, its impact on meeting cellular demand has not been addressed. Such disparity of RBCs observed in the capillaries could have an important role in the alteration of blood viscosity and velocity as part of the Fåhraeus effect. This in turn could enable a smoother and more efficient passage for blood moving through the vessels to satisfy changing cellular demands. Our findings in the retinal microvasculature from a living preparation support the presence of the Fåhraeus effects.

Several limitations are noted in the current study. Firstly, the anaesthetics mix of medetomidine and ketamine used in this study is known to have an impact on regional cerebral blood flow in cats^13^. However, it is not known whether the retinal fRBC flow in the rat would be similarly affected. Secondly, blood glucose was notably elevated in the rats in the current study, likely a consequence of medetomidine use^17^. Whilst acute hyperglycemia is reported to reduce the cerebral blood flow in mice^31^, increased retinal blood flow was reported in cats^32^. The impact of acute hyperglycaemia on retinal fRBC flow in the rat is unclear and an important consideration in future investigations. Thirdly, whilst the power level of the 640 nm line (< 741 µW) is well below the recommended level (0.39mW) for thermal effect, the power level of the 488 nm laser used (< 120 µW) is above the recommended exposure limit (39 µW) for photochemical effect. It is unclear whether the 488 nm power level has initiated a photochemical effect and if the current level of laser exposure has affected the retinal fRBC flow.

Inherent heterogeneity exists within the structural geometry of the vascular network as reflected in the intraretinal heterogeneity of retinal oxygen distribution^33–38^. The presence of spatial variation in fRBC speed and distribution in the different vessel segments and the different layers of the retina within the same rat in the current study is also reflective of such inherent heterogeneity. Whilst we cannot eliminate the possible effect of laser or anaesthetic exposure on the retinal blood flow, the dynamic temporal variation observed in fRBC speed is likely an inherent property of retinal blood flow because the rats were breathing room air and not exposed to additional metabolic challenges. This dynamic temporal variation is likely associated with the downstream transmission of systemic pulsation^39^ though we were not able to confirm this association with the current acquisition parameter settings. A much higher frame rate together with a greater proportion of fRBCs for measurement are needed to investigate this in future studies. The dynamic nature of the blood flow may also be an important point to note when considering how the retinal microvasculature may autoregulate within its hierarchical structure in response to changing neuronal demands.

In the short-term, regulation of blood flow in the microvascular network is known to occur by myogenic response to neural, hormonal, metabolic, autacoidal (secreted) and mechanical factors. We have investigated the possible contribution of temporal variation in vessel diameter in relation to the observed temporal variation in fRBC speeds in the larger arterioles. However, the arteriole diameters appear to stay consistent through the time frames (data not shown) and no obvious association was noted.

Many aspects are known to contribute to the flow properties within the microcirculation. Other than the inherent anatomical heterogeneity of the vascular segments with different diameters, different sensing and regulatory mechanisms are present in the arterioles, capillaries and venules. The cellular component within blood such as red blood cells, white blood cells, platelets and their deformability and adhesiveness to the vessel wall could influence the viscosity and fluidity of blood flow through the small vessels in the microcirculation. Much of the published work in the retina have relied on detecting changes in diameter from single vessels^18, 40^, mostly from the major retinal arterioles and veins, and using this to infer the change in blood flow in the capillary network. Many in vitro experiments have been conducted along with mathematical modelling and sophisticated theoretical analyses of blood flow in an attempt to characterise capillary network flow^41, 42^, although retinal vessels ex-vivo are known to respond differently to metabolic challenges than in vivo^43^. One major advantage of the current study is the ability to measure red blood cell velocity from the retinal capillary network at different depths within the retina using in vivo time-series. More recent work using adaptive optics^44^ have found unexpected but significant changes in diameter in the pre-capillary arterioles, capillaries and post-capillary venules in the functional hyperaemia response in the human retina, pointing to a much more dynamic redistribution of retinal flow in response to local, stimulus-driven metabolic demand. However, the extent of the spatial flow redistribution and the interplay between the various mechanisms is yet to be elucidated. The current technique can contribute through simultaneous in vivo investigation of multiple vessels within the retinal network (compared to previous studies based on single vessel) for the dynamic flow redistribution.

The retina, being an unusually large and thin sheet of neuronal tissue with a compact and highly organized neuronal layer structure, has the highest metabolic demands in the body. Constant stimulation from light triggers a highly dynamic metabolism requiring support from a continuously regulated microcirculation. Although it is known that different sensors and regulatory mechanisms are present at different levels of the microcirculation, how the different mechanisms are coordinated in a concerted fashion for a dynamic redistribution of flow in response to physiological and pathological challenges remains to be elucidated. The current study has identified that the in vivo retinal capillary network fRBC speeds are spatially and temporally dynamic in nature. This observation is consistent with the presence of spatial and temporal heterogeneity in retinal capillary perfusion postulated^45^ and identified in human macular capillaries in OCTA images^28^. It is envisaged that the interplay between neuronal metabolic demands, systemic and local hormonal responses, vascular myogenic and autocoidal factors will be complex. The current technique could be applied to study the dynamic changes in fRBC flow speed at the different levels of the retinal microvascular network to further this understanding. Given the link between retinal pathology and other neurological and systemic diseases, further understanding of how retinal microvessels respond and react to metabolic and pathologic challenges could help elucidate the mechanism of microvascular dysfunction in systemic illnesses.

## Materials and methods

### Ethics consideration

The study was approved by The University of Western Australia Animal Ethics Committee. All animal procedures conformed to the Australian Code for the care and use of animals for scientific purposes. The authors complied with the ARRIVE guidelines^46^.

### Animal preparation

**Figure 6:**
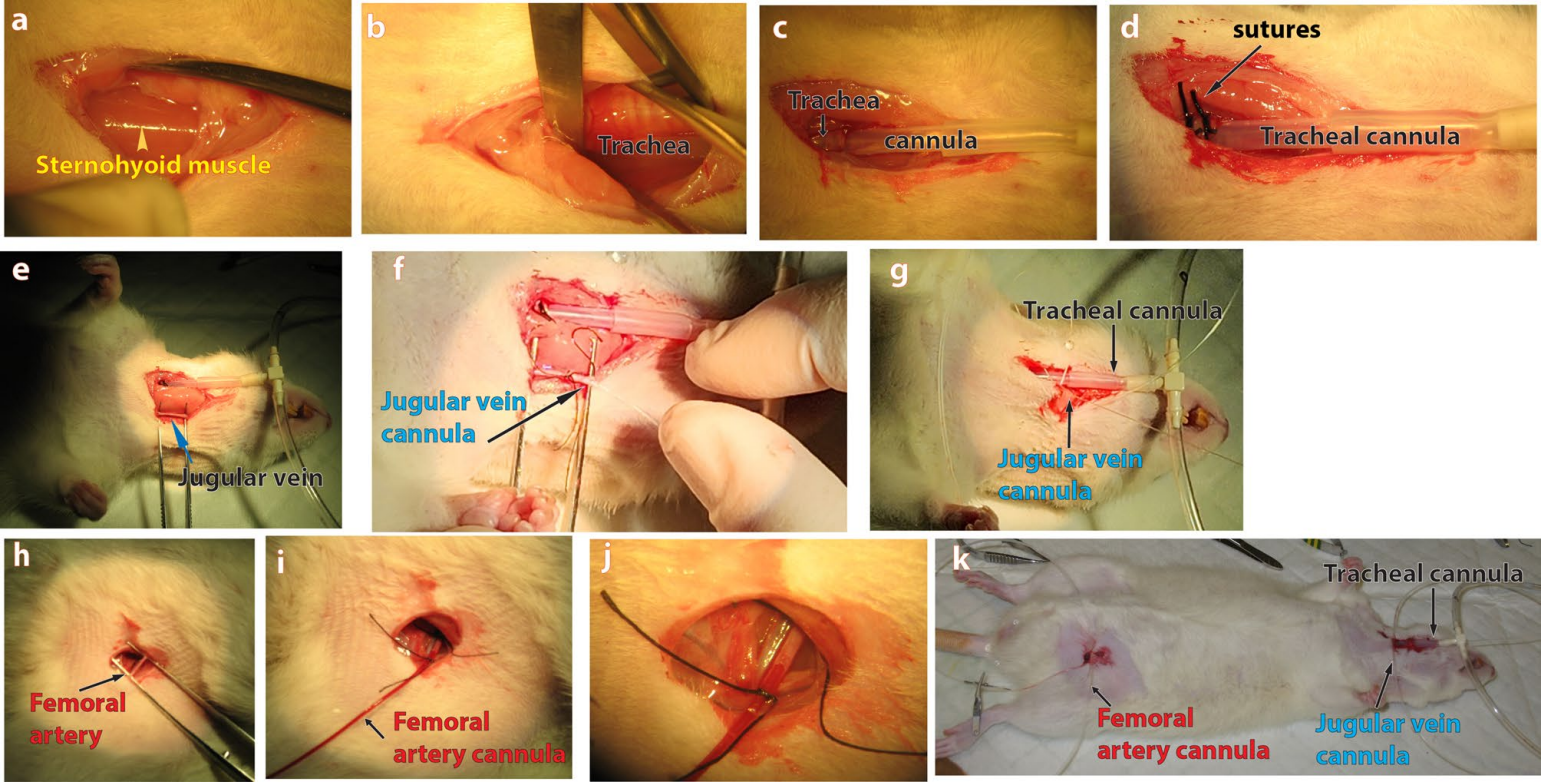
Surgical procedures to prepare rat for in vivo imaging. (**a**)–(**d**) shows the steps involved in tracheotomy, (**e**)–(**g**) shows the steps involved in the jugular vein cannulation, and (**h**)–(**k**) shows the steps involved in femoral artery cannulation. (**a**) shows a ventral cervical incision in the throat region. The skin, subcutaneous fat has been cut to expose the sternohyoid muscle (yellow arrowhead). (**b**) shows retraction of the sternohyoid muscle pair to expose the trachea lying underneath. (**c**) shows the trachea with an incision made and a cannula inserted into the tracheal lumen. (**d**) shows the tracheal cannula securely tied to the tracheal using two 3.0 sutures. (**e**) shows the exposure of the left jugular vein by gross dissection. (**f**) shows the jugular vein being cannulated and two double looped 5.0 sutures for securing the inserted cannula to the vein. (**g**) shows the throat region with a 4.0 suture closing the skin incision after the jugular vein cannula and tracheal cannula have been securely inserted. (**h**) shows the exposure of the femoral artery in the left inguinal region of this rat after gross dissection to free it from the femoral neurovascular sheath. (**i**) shows the femoral artery successfully cannulated with blood flowing into the cannula. The femoral artery cannula has been tied to the femoral artery using two double looped 5.0 sutures. (**j**) is a magnified view of the cannula going into the femoral artery. (**k**) shows the ventral side of the rat after the tracheal, jugular vein and femoral artery cannulas have been successfully inserted. Note that the fur in the surgery region had been shaved prior to surgery.

Eight young adult male Sprague Dawley rats at 15 weeks of age with an average weight of 530 ± 47.4 g were used. Anaesthesia was induced by an intraperitoneal (i.p.) injection of 50 mg/kg Ketamine (100 mg/ml) and 10 mg/kg Medetomidine (1 mg/ml) and maintained every 40 min with a half dose injection. Surgery was performed once the rat was unresponsive to foot pinch. Tracheotomy (Fig. 6a–d) was performed to enable artificial ventilation using room air initially at 60–80 breaths per minute at 2.5 ml tidal volume. One jugular vein (Fig. 6e–g) was cannulated for injection of FITC-dextran and labelled red blood cells and a femoral artery was cannulated (Fig. 6h–k) for continuous monitoring of blood pressure, heart rate and blood sampling. The vessels cannulation steps are similar to those previously reported^47, 48^. Blood samples were obtained and used for evaluation and maintenance of blood gas, pH and blood glucose within normative range^49^ through adjustments of breathing rate and tidal volume. Ventilation rate and volume were adjusted until the blood pH fell in the range of 7.35–7.45, and blood CO_2_ fell between 35 and 45 mmHg before the start of experiment. For most rats, the ventilation rate of 80–90 breaths per minute at a 3.5 ml tidal volume was used.

### Experiment set-up and protocol

The rat was placed on a temperature regulated platform and the head held securely in place using a custom-made head holder and ear bars (Fig. 1a). The head holder enables axial turning of the rat head to orientate the eye to the downward positioned objective lens for confocal imaging.

Between 410 and 800 µl of blood was drawn from the femoral artery line for labelling using the lipophilic tracer DiD (D7757, InvitrogenTM from ThermoFisher Scientific, Australia). The labelling protocol is detailed in Kornfield and Newman’s paper^18^. The labelled fRBC were made up to 1 ml in blood plasma buffer and injected gradually back into the rat via jugular vein cannula. 1 ml of FITC-Dextran (2000 kDa; 3% in saline; Sigma FD2000S) was injected slowly into the rat via the jugular vein cannula and any residue FITC-dextran left in the line washed into the rat using 1 ml of heparinised saline. Throughout the injection of FITC-Dextran, the blood pressure was closely monitored to avoid a sudden increase or drop in blood pressure.

As soon as the blood pressure had stabilised with the rat breathing room air and blood pH and blood gases confirmed to be within the normative range, in vivo imaging took place, and several time series videos were captured from the superficial and deep retinal vascular plexus of the rat eye. Generally, one hour was required to complete the surgical procedures and another 40 min for the red blood cells labelling. Depending on the length of time required to adjust the respiratory parameters for the rat so that the blood gas readings were within the normative range, in vivo imaging took place from 2.5 to 4 h after the initial induction of the anesthesia.

### In vivo imaging

A Nikon A1R system equipped with a CFI Plan Fluor 4x (NA = 0.20; WD = 20.00 mm) and a 10 × dry lens (NA = 0.30; WD = 16.0 mm), laser lines 488 nm and 640 nm wavelengths was used for the data acquisition on NIS-Elements AR (version 5.30.05). The rat cornea was protected from dehydration with a drop of Healon and a clear semi-concave contact lens. Time series were acquired using the 488 nm and 640 nm laser lines coupled with detection at 525/50 nm (frequency/bandwidth) and 700/75 nm respectively for FITC-dextran (Ex 490 nm, Em 520 nm) and DiD (Ex 644 nm, Em 665 nm) signal. In all cases the cornea and lens remained visibly clear throughout the experiment at the laser settings used (< 120 µW for 488 nm line and < 741 µW for 640 nm line). The superficial and deep retinal vessels were identified based on topography and the focal plane fine adjusted by viewing the live images on the monitor.

Separate image time series of a single optical section (> 30 µm) were captured with the 10 × dry lens using resonance mode for the purpose of either capturing fRBC movement or making vessel diameter measurements. In the former case, 2-channel images (Cy5 and FITC) were captured at ~ 4.93 µm per pixel with NIS-Elements live denoising and deconvolution at approximately 58.25 frames per second. For the latter, single-channel images (FITC) were captured at ~ 1.23 µm per pixel resolution at 15.23 frames per second without any live processing. Image series were acquired consecutively for the SL and DL.

#### Image pre-processing: Denoising and spatial alignment

NIS-Elements was used to independently denoise and smooth each channel of each frame. For each time series in turn, NIS-Elements was used to align each frame to the first frame based on the FITC channel.

### Histology preparation and confocal imaging

At the conclusion of the experiment the rat was euthanized with an overdose of Pentobarbital (160 mg/kg) and the eyes immediately enucleated for histology.

The isolated eye imaged using in vivo microscopy was cannulated at the ophthalmic artery for perfusion fixation and intravascular labelling within an hour of enucleation. The perfusion protocol is similar to that used in a previous study of human donor eyes and isolated porcine eyes^50–53^. Due to the intrascleral branching of the rat ophthalmic artery to supply the choroid and the retina, all vortex veins had to be ligated on the rat eye to enable full retinal perfusion. The eye was perfused at 110 µl per minute with heparinised Ringers (10 min), then 4% paraformaldehyde in 0.1 M phosphate buffer (PB) (20 min), 60 µg Lectin-FITC (L4895, Sigma-Aldrich, Merck Australia; 2 boluses at 16 min interval) in PB, followed by a 12-min wash with PB, 7-min wash with 1% Triton-X-100 in PB, 2 µg Phalloidin TRITC in PB (P1951, Sigma-Aldrich, Merck Australia; 3 boluses of 160 µl at 20 min interval) and a final 15-min PB wash. The retina was then carefully dissected out, mounted in RapidClear®1.47 (Sunjin Lab., Taiwan), and cover-slipped for confocal imaging.

A few eyes were immunolabelled by intravascular perfusion for α-SMA. The protocol of perfusion is as described above but replacing the Phalloidin step with a mixture of anti-αSMA (A2547 Sigma-Aldrich; 1:25 dilution) /goat serum (10%) / 0.1% Triton-X-100 / Hoechst. The retina was then floated in secondary antibodies (goat anti-mouse conjugated to Alexa Fluor 555, Abcam ab150114; 1:200) / 0.1% Triton PB / Hoechst 33,342 (Merck 94403; 1:1000) overnight. It was subsequently washed in 0.1 M PB for a day and then flat mounted in RapidClear®1.47 (Sunjin Lab Co., Taiwan) for confocal microscopy.

Labelled retinal microvasculature was imaged on a Nikon C1 plus confocal system using the Plan Apo 20 × objective lens (NA = 0.75; WD = 1.0 mm). The entire retinal microvasculature was perfuse labelled using Lectin-FITC, which binds to the endothelial glycocalyx, and imaged using the 488 nm laser line. Phalloidin-TRITC and αSMA immunolabeling, enabling visualisation of vascular smooth muscle cells, were imaged using the 546 nm and 640 nm laser lines. Hoechst counterstaining for nuclei was imaged using the 405 nm UV laser. A stack of optical sections (2D images) of the flat mounted rat retina was acquired with a step size (z-dimension) of 1 µm and a pixel size of 0.31–0.62 µm^54^. Figure 1b shows a maximum intensity z-projection through one of the acquired stacks and depth coded from SL and DL.

### Selection and categorisation of vessel segments for analysis

**Figure 7:**
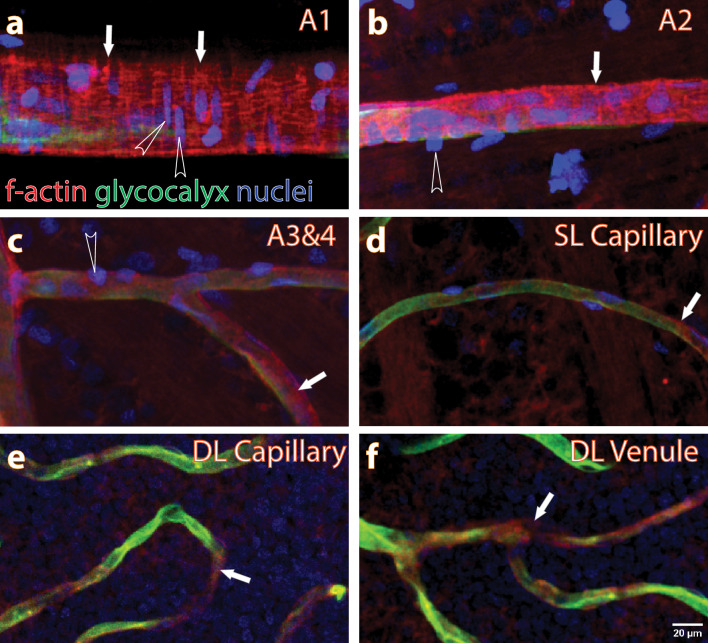
Categorization of vessel segments. The retinal microvasculature was perfuse labeled for glycocalyx (green, using Lectin-FITC), f-actin (red, using phalloidin) and nuclei (blue, using Hoechst). (**a**–**d**) are histology projection images of A1, A2, A3, A4 and capillary vessel segments from the SL. (**e** and **f**) are capillary and venule segments from the DL. In all the projected images of the vessel segments, f-actin labeling (white arrows) a differing pattern of arrangement may be seen on the outside of vessel segments. F-actin filaments are densely populated around the A1 and A2 vessel segments in a circular pattern alongside the nuclei of vascular smooth muscle cells (open arrowhead). F-actin filaments are also present around A3, A4, capillaries and venule segments, with a reduced density and regular pattern of appearance. Scale bar in (**f**) applies to (**a**–**f**).

The histology images were used to select vessel segments (curvilinear paths/trajectories) in the SL and DL for analysis. From each vascular layer of each rat, at least 10 vessel segments were selected for analysis of vessel diameter (both in histology and in vivo), fRBC velocity and fRBC counts. Vessel segments were categorised according to the presence and appearance of vascular smooth muscle cells (Fig. 7), branching order and diameter. Vessel segments branching directly from a major radiating retinal artery with circularly arranged smooth muscle cells were categorised as A2, branches from A2 were categorised as A3, and branches from A3 were categorised as A4. A vessel segment was categorised as a capillary, C, when no smooth muscle cells could be seen wrapping around it. The first convergence of capillaries was categorised as V3, convergence of V3 vessel segments was categorised V2, and V2 vessels were observed to drain directly into one of the radiating retinal veins. Some capillaries that were observed to drain directly into the major retinal vein were categorised Vc.

### Image analysis

Image analysis was performed in FIJI^55^ using the MorphoLibJ^56^ library and the CLIJ2 GPU-accelerated image processing library^57^, and in R^58^ using the pracma package^59^. Custom scripts were written to compute fRBC velocity measurements using kymograph analysis (Fig. 2), to detect fRBCs in each frame of an in vivo time series to compute fRBC counts, and to compute vessel diameter measurements on the in vivo data.

#### Computation of vessel segment fRBC velocity

For each fRBC image series (SL and DL) in turn, FIJI was used to visualise the flow direction, compute the pixel-wise standard deviation for the Cy5 channel to visualise the fRBC tracks, and to draw curvilinear ROIs (segmented lines) corresponding to the vessel segments selected from histology (Figs. 2b and 3a,c). The ROIs were drawn along the centreline of the respective vessel segment from upstream to downstream in the direction of fRBC flow (Figs. 2b and 3a,c). The speed (velocity magnitude) for each vessel segment was computed using the FIJI script "compute_kymograph_direction_histograms.ijm" (Software S1) and R script "kymo_analysis.R" (Software S2).

The FIJI script constructs a kymograph (space–time plot) for each vessel segment. Each kymograph is a 2D image with the first row containing the intensities along the vessel segment line ROI for the first frame, the second row containing those from the second frame and so on (Fig. 2c). Stationary fRBCs appear as bright vertical streaks, whilst moving fRBCs appear as diagonal streaks. For each kymograph the script uses FIJI’s Directionality plugin (Fourier power spectrum approach) to generate a histogram indicating the amount of structure at each angle (Fig. 2d). The R script fits a natural cubic spline to smooth the histogram. It then identifies the peak, which corresponds to the slope of the dominant (most frequently occurring) streaks in the kymograph, and uses this to compute fRBC speed (Fig. 2e).

#### Computation of vessel segment fRBC counts (proxy measure of haematocrit)

For each fRBC image series (SL and DL) in turn, the FIJI script "spot_detector.ijm" (Software S3) was used to detect fRBC candidates in each frame. The script first applies a Laplacian of Gaussian filter of size ~ 6.5 µm (diameter of a rat RBC) to suppress the background and enhance the contrast of fRBC-sized spots. It then permits the user to interactively select spots in the first frame in terms of prominence (FIJI’s Find Maxima tool) and mean intensity. These two parameters are chosen such that spots likely to be from fRBCs in vascular layers above or below the focal layer of interest are suppressed. The chosen parameters are then applied to all frames to generate a binary stack in which each fRBC is indicated with a binary 1. Each frame of this stack is then dilated to form a second binary stack where each fRBC has the approximate size of a rat RBC (Fig. 4d). This stack can then be used to generate a plot of vessel segment fRBC counts over time (Fig. 4e) and fRBC count maps i.e., pixelwise fRBC counts over time (Fig. 4f–i).

#### Vessel diameter measurement

For each histology image stack, FIJI was used to create maximum intensity projections through the SL and DL respectively. FIJI’s line tool was then used to measure each of the selected vessel segments in the SL and DL.

Vessel diameters were also measured (SL and DL) for each single channel FITC in vivo image series^60^. For each series, a 30 s sequence was selected and every 100th frame (i.e., every 6.6 s) extracted for measurement. The FIJI script "vessel_diameter.ijm" (Software S4) was used to draw a line ROI perpendicular to the longitudinal axis of each vessel of interest on the average frame, and to estimate the diameter of each vessel in each frame by computing the full width at half maximum (FWHM) of the intensity profile across the corresponding line ROI using the implementation of McDowell et al.^61^.

### Statistical analysis

Statistical analysis was performed in R using the packages nlme^62^ to fit linear mixed effects models; emmeans^63^ to compute estimated marginal means (predictions) from the models, and to perform pairwise comparisons; and rmcorr^64^ to compute repeated measures correlation. All hypothesis tests were performed at the 5% level of significance.

#### Statistical analysis of fRBC speed

To investigate the difference in fRBC speed between the SL and DL whilst accounting for variability among rats, a linear mixed effects model (LMM) was fitted to the speed data. The model was fitted with fRBC speed as the response variable, layer and vessel type as fixed effects, and rat ID as a random effect. To satisfy model assumptions (normally distributed residuals with constant variance) it was necessary to log transform the response (Shapiro–Wilk test, p = 0.1617). The model was also used to estimate marginal means and their standard errors (SEs) for each vessel type and layer.

#### Statistical analysis of vessel diameter

A LMM was similarly fitted to the vessel diameter data with diameter as the response variable; measurement method (either in vivo or histology), layer and vessel type as fixed effects; and vessel type nested in rat ID as the random effects structure. To satisfy model assumptions it was necessary to transform the response variable by applying a Box-Cox transform with λ = − 0.3 (Shapiro–Wilk test, p = 0.06052). The model was used to estimate marginal means and their SEs for each vessel type and layer for each measurement method.

#### Statistical analysis of fRBC speed and vessel diameter

To investigate whether vessel diameters measured in vivo or from histology were predictive of fRBC speed measurements, a LMM was fitted to the combined speed and diameter data with fRBC speed as the response variable. The most parsimonious model included layer and histology vessel diameter as fixed effects, and rat ID as the random effect. To satisfy model assumptions it was necessary to log transform the response (Shapiro–Wilk test, p = 0.6251). Repeated measures correlation^65^ was used to compute the correlation between histology vessel diameter and fRBC speed for both layers, and each layer separately. The method accounts for the non-independent observations (multiple paired measurements for each rat) which may produce biased, specious correlation estimates if simple regression / correlation is used^65^.

### Supplementary Information

Supplementary Legends.

Supplementary Video 1.

Supplementary Information 2.

Supplementary Information 3.

Supplementary Information 4.

Supplementary Information 5.

Supplementary Information 6.

## Data Availability

The datasets generated during the current study are available in the UWA Profiles and Research Repository [https://doi.org/10.26182/rftm-5q12, https://doi.org/10.26182/b5x9-x211, https://doi.org/10.26182/6q1t-vz55]. The FIJI and R scripts used for image analysis can be found in the supplementary material online.
