# Supplementary material for: Quantitative study of spatial and temporal variation in retinal capillary network perfusion in rat eye by in vivo confocal imaging
Source: Sci Rep. 2023 Nov 2;13:18923. doi: 10.1038/s41598-023-44480-1 (PMC10622421; doi:10.1038/s41598-023-44480-1)
Supplement: Supplementary file 1 — Supplementary Legends. [file 41598_2023_44480_MOESM1_ESM.pdf]

Quantitative study of spatial and temporal variation in retinal capillary network perfusion in rat eye by in vivo confocal imaging

Authors:
Paula Kun Yu[1,2]
Andrew Mehnert[1,2]
Jayden Brendan Dickson[2]
Hassanain Qambari[1,2]
Chandrakumar Balaratnasingam[1,2,3]
Stephen Cringle[1,2]
Dean Darcey[2]
Dao-Yi Yu[1,2*]

[1]Centre for Ophthalmology and Visual Science, The University of Western Australia, Perth, Australia
[2]Lions Eye Institute, 2 Verdun St, Nedlands, WA, Australia
[3]Department of Ophthalmology, Sir Charles Gairdner Hospital, Western Australia, Australia

***Corresponding Author:**
Professor Dao-Yi Yu
Lions Eye Institute,
2 Verdun Street, Nedlands, WA, Australia
Email: dao-yi.yu@uwa.edu.au

Supporting Information File

Movie S1 (Movie S1.avi). Movie showing a representative 30 second FITC and Cy5 channel in vivo time series after preprocessing and alignment. The pixel size is 4.93µm and the original frame rate was 58.25 fps. The frame rate of the movie has been reduced to 15 fps for easier visualisation of fRBC movement.

Software S1 (compute_kymograph_direction_histograms.ijm). FIJI script used in the computation of vessel segment fRBC velocity.

Software S2 (kymo_analysis.R). R script used in the computation of vessel segment fRBC velocity.

Software S3 (spot_detector.ijm). FIJI script used to detect fRBC candidates in each frame of an in vivo time series.

Software S4 (vessel_diameter.ijm). FIJI script used to estimate selected vessel diameters in each frame of an in vivo time series.

*The scripts Software S1-S4 are presented with syntax highlighting in **Software S1-S4.docx**.
