# Supplementary material for: Quantitative study of spatial and temporal variation in retinal capillary network perfusion in rat eye by in vivo confocal imaging
Source: Sci Rep. 2023 Nov 2;13:18923. doi: 10.1038/s41598-023-44480-1 (PMC10622421; doi:10.1038/s41598-023-44480-1)
Supplement: Supplementary file 7 — Supplementary Information 6. [file 41598_2023_44480_MOESM7_ESM.docx]

# Software S1

## compute_kymograph_direction_histograms.ijm

*/**
 ** Author: Andrew Mehnert, Centre for Ophthalmology & Visual Science, UWA*
 ***
 ** SYNOPSIS:*
 ***
 ** History: 10/8/2002 - V1.0*
 ***
 **/*


setOption("JFileChooser", **true**)
run("Close All");


**function** replace_spaces_with_underscore(string) {
 fixed_string = replace(string, " ", "_");
 **return** fixed_string;
}

*// Ask user to select the nd2 file to process*
filepathname = File.openDialog("Select the RBC \"Aligned.nd2\" file to process");
filename_without_extension = replace_spaces_with_underscore(File.getNameWithoutExtension(filepathname));
directory = File.directory;

*// Load as virtual stack*
run("Bio-Formats","open=[/"+filepathname+"] autoscale color_mode=Default rois_import=[ROI manager] view=Hyperstack stack_order=XYCZT use_virtual_stack");
rename("input");

*// Load the user-specified number of slices as a stack*
waitForUser("Info", "Please review the stacks to decide how many frames you want to use");
slices_to_use = getNumber("How many frames to use?", nSlices);
run("Make Substack...", "slices=1-"+slices_to_use);
run("Enhance Contrast...", "saturated=0 normalize process_all use"); *// linearly stretch intensities over entire bit range*
close("input");
rename("input");

*// Get pixel size and frame rate from the metadata*
Stack.getUnits(X_unit, Y_unit, Z_unit, Time_unit, Value_unit);
getPixelSize(unit, pixelWidth, pixelHeight);
frameInterval = Stack.getFrameInterval(); *// in Time_unit*
frameRate = 1/frameInterval; *// in 1/Time_unit*
pixelSize = pixelWidth;
number_of_frames = nSlices;
total_time = number_of_frames * frameInterval;
showMessage("Properties for the stack", "Frame rate = "+frameRate+" per "+Time_unit+"\n"
 +"Frame interval = "+frameInterval+" "+Time_unit+"\n"
 +"Pixel size = "+pixelSize+" "+X_unit+"\n"
 +"Units of speed are "+X_unit+" per "+Time_unit+"\n"
 +"Total time = "+total_time+" "+Time_unit);

*// Create a stDev z-projection to visualise the RBC pathways (i.e. vessels)*
run("Z Project...", "projection=[Standard Deviation]");
run("Enhance Contrast...", "saturated=0.35 normalize");
run("Enhance Local Contrast (CLAHE)", "blocksize=127 histogram=256 maximum=3 mask=*None*");
setOption("ScaleConversions", **true**);
run("16-bit");

*// Duplicate this as many times as there are frames in the RBC stack*
selectWindow("STD_input");
run("Copy");
**for** (i = 2; i <= number_of_frames; i++) {
 run("Add Slice");
 run("Paste");
}

*// Create colour composite*
run("Merge Channels...", "c1=input c2=STD_input create");
rename("input");
run("Re-order Hyperstack ...", "channels=[Channels (c)] slices=[Frames (t)] frames=[Slices (z)]");
Stack.setChannel(1);

*// Load or create line segment ROIs for kymograph analysis*
setTool("polyline");
waitForUser("Load or define line segment ROIs and click OK when done");

*// For each ROI, generate a kymograph, compute its direction histogram and write this to the "Kymograph direction histograms" table*
n = roiManager('count');
setBatchMode("hide");
Table.create("Kymograph direction histograms");
run("Split Channels");
selectWindow("C1-input");

**for** (i = 0; i < n; i++) {
 selectWindow("C1-input");
 roiManager('select', i);
 name = Roi.getName;

 run("Reslice [/]...", "output=1.000 start=Top avoid");
 rename("Kymograph of "+name);

 directionAndFit = eval("script",
 "analyzer = new " + "Packages.fiji.analyze.directionality.Directionality_()" + ";"
 + "analyzer.setImagePlus(Packages.ij.IJ.getImage());"
 + "analyzer.computeHistograms();"
 + "analyzer.fitHistograms();"
 + "table = analyzer.displayResultsTable();"
 + "table.show(\"Directionality histogram\");");

 *//close("Kymograph of "+name);*
 table_heading = split(Table.headings("Directionality histogram"),"\t");
 **if** (i==0) {
 direction = Table.getColumn(table_heading[0],"Directionality histogram");
 Table.setColumn("Direction", direction, "Kymograph direction histograms");
 }
 rel_freq = Table.getColumn(table_heading[1],"Directionality histogram");
 Table.setColumn(name, rel_freq, "Kymograph direction histograms");
 Table.update("Kymograph direction histograms");
 close("Directionality histogram");
}
setBatchMode("exit and display");

run("Merge Channels...", "c1=C1-input c2=C2-input create");

*// Add some additional metadata to the table and save*
Table.set("Number of frames", 0, number_of_frames, "Kymograph direction histograms");
Table.set("Frame interval ("+Time_unit+")", 0, frameInterval, "Kymograph direction histograms");
Table.set("Frame rate (per "+Time_unit+")", 0, frameRate, "Kymograph direction histograms");
Table.set("Pixel size ("+X_unit+")", 0, pixelSize, "Kymograph direction histograms");
filepathname = directory+filename_without_extension+"_("+slices_to_use+"_slices)_kymo_direction_histograms.csv";

Table.save(filepathname,"Kymograph direction histograms");
showMessage("Info","Table written to "+filepathname);

# Software S2

## kymo_analysis.R

#

# Author: Andrew Mehnert, Centre for Ophthalmology & Visual Science, UWA

#

# SYNOPSIS: Processes one or more "..kymo_direction_histograms.csv" files

# produced by "compute_kymograph_direction_histograms.ijm".Each

# CSV file contains the following columns: Direction, ROI1, ROI2, ...,

# Number of frames, Frame interval (sec), Frame rate (per sec),

# Pixel size (micron). The ROI columns contain the relative frequency

# counts for each direction. For each file:

# 1. The dominant directions either side of zero degrees are determined

# for each ROI, and used to compute the forward and backward speeds

# 2. The speeds are written to "...speed_table.csv"

# 3. Plots of the histogram of directions for each ROI are written to a multipage PDF

#

#

#

# History: 10/08/2022 - V1.0

# 11/08/2022 - V1.1 - Plot now automatically saved as an SVG file

# 30/08/2022 - V1.2 - Removed SVG output and instead plot to a PDF file

# 19/09/2022 - V2.0 - Modified so that all files of the

# form "*_kymo_direction_histograms.csv" are

# processed in a specified directory

# 13/02/2023 - V2.1 - Modified plots to show x-axis labels from -90 to 90 in increments of 10

#

library**(**pracma**)**

library**(**svDialogs**)**

# Ask user to select a directory containing the CSV files to process

answer **=** dlg_dir**(**default **=** "", title **=** "Choose the directory containing one or more \"*kymo_direction_histograms.csv\" files to be processed"**)**

src_dir **<-** answer**$**res

# Create a list of the CSV files to be processed

csvfiles **<-** list.files**(**src_dir, pattern**=**'_kymo_direction_histograms.csv$', recursive**=FALSE**, full.names **=** **TRUE)**

# Process each file in turn

**for** **(**i **in** 1**:**length**(**csvfiles**)){**

filepathname **<-** csvfiles**[**i**]**;

print**(**paste**(**"################# Processing file", filepathname**))**

histogram **<-** read.table**(**filepathname,header**=TRUE**,sep**=**",",stringsAsFactors **=** **FALSE)** # Read in the CSV file

pixel_size **=** histogram**$**Pixel.size..micron.**[**1**]**

frame_interval **=** histogram**$**Frame.interval..sec.**[**1**]**

x **<-** histogram**$**Direction

number_of_ROIs **<-** ncol**(**histogram**)** **-** 5;

forward_speed **<-** vector**()**;

backward_speed **<-** vector**()**;

plotfilepathname **<-** file.path**(**dirname**(**filepathname**)**,paste0**(**strRep**(**basename**(**filepathname**)**,".csv",".pdf"**)))**

pdf**(**file **=** plotfilepathname**)**

par**(**mfrow**=**c**(**2,2**))**

finer_x **<-** seq**(**from**=-**89, to**=**89, by **=** 0.5 **)**

**for** **(** ROI_count **in** 1**:**number_of_ROIs**)** **{**

y **<-** histogram**[**,1**+**ROI_count**]**

**if** **(**any**(**is.nan**(**y**)))** **{**

print**(**paste**(**"ROI",ROI_count,"contains NaN values. ROI has been excluded from the speed table"**))**

plot**(**finer_x,rep**(**0,length**(**finer_x**)))**

**next**

**}**

f_of_x **<-** splinefun**(**x,y,method**=**'natural'**)**

fitted_y **<-** f_of_x**(**finer_x**)**

result**<-** findpeaks**(**fitted_y,sortstr **=** **TRUE)**

locn **<-** result**[**,2**]**

peak_value **<-** result**[**,1**]**

plot**(**finer_x,fitted_y,type**=**"l", xlab **=** "Direction (degrees)", ylab **=** "Relative frequency", xaxt **=** 'n'**)** #, main=paste0("ROI",ROI_count))

axis**(**1, at **=** seq**(-**90, 90, by **=** 10**)**, las**=**2**)**

#lines(finer_x[locn],peak_value,type="p")

# Constant forward speed (i.e. in the direction from the start to the end of a vessel track)

# generates downward sloping lines in the kymograph. To compute this we need to

# determine the most dominant negative direction

location_of_neg_angle_with_largest_peak **<-** locn**[**which**(**finer_x**[**locn**]<=-**1**)[**1**]]**

dominant_direction **<-** finer_x**[**location_of_neg_angle_with_largest_peak**]**

lines**(**dominant_direction,fitted_y**[**location_of_neg_angle_with_largest_peak**]**,type**=**"p",col**=**"red"**)**

#print(dominant_direction)

tan_theta **=** tan**(**abs**(**dominant_direction**)***pi**/**180**)** # tan of the angle gives #time_pixels divided by #dist_pixels in the kymograph

forward_speed**[**ROI_count**]** **=** 1**/**tan_theta ***** pixel_size **/** frame_interval # speed is (#dist_pixels * pixel_size) divided by (#time_pixels * frame_interval)

#print(forward_speed[ROI_count])

# Constant backward speed generates upward sloping lines in the kymograph

location_of_pos_angle_with_largest_peak **<-** locn**[**which**(**finer_x**[**locn**]>=**1**)[**1**]]**

dominant_direction **<-** finer_x**[**location_of_pos_angle_with_largest_peak**]**

#lines(dominant_direction,fitted_y[location_of_pos_angle_with_largest_peak],type="p",col="red")

#print(dominant_direction)

tan_theta **=** tan**(**abs**(**dominant_direction**)***pi**/**180**)**

backward_speed[ROI_count] = 1/tan_theta * pixel_size / frame_interval

#print(backward_speed[ROI_count])

}

speed_table <- data.frame(colnames(histogram)[2:(number_of_ROIs+1)],forward_speed,backward_speed)

tablefilepathname <- file.path(dirname(filepathname),paste0(strRep(basename(filepathname),"_kymo_direction_histograms","_speed_table")))

# write.table(speed_table, tablefilepathname, sep=",", row.names = FALSE)

dev.off()

print(paste("Speed table written to",tablefilepathname))

print(paste("Plots written to", plotfilepathname))

}

# Software S3

## spot_detector.ijm

*/**
 ** Author: Andrew Mehnert, Centre for Ophthalmology & Visual Science, The University of Western Australia*
 ***
 ** History: 31/8/2022: V1.0*
 ***
 **/*


*// User-defined constants*
RBC_size_in_microns = 6.5; *// size of rat RBC*
default_prominence_for_maxima_detection = 0.01;

*// Close any open windows and the ROI Manager*
close("*");
close("ROI Manager");

*// Ask user to open the stack to process*
waitForUser("Open or drag-and-drop the fRBC stack to process and click OK to continue");
image_name = getInfo("window.title");

*// Ask user for the range of frames to use and create substack*
slices_to_use = getString("Enter the range (e.g. 2-550) of frames that you want to use", "1-"+nSlices);
run("Make Substack...", "slices="+slices_to_use);
number_of_slices = nSlices;

*// Constrast enhance*
run("Enhance Contrast...", "saturated=0.35 normalize process_all use"); *// linearly stretch intensities over entire bit range*
close(image_name);
rename(image_name);

*// Get pixel size and frame rate*
Stack.getUnits(X_unit, Y_unit, Z_unit, Time_unit, Value_unit);
getPixelSize(unit, pixelWidth, pixelHeight);
frameInterval = Stack.getFrameInterval(); *// in Time_unit*
frameRate = 1/frameInterval; *// in 1/Time_unit*
pixelSize = pixelWidth;
total_time = nSlices * frameInterval;

*// Calibrate filter sizes*
RBC_size_in_pixels = RBC_size_in_microns/pixelSize;
radius_of_mean_filter_in_pixels = Math.ceil((RBC_size_in_pixels - 1)/2); *// radius in pixels such that 2*radius+1 pixels COVER a single RBC*
radius_of_LoG_filter_in_pixels = radius_of_mean_filter_in_pixels + 1;

showMessage("Properties for the stack", "Frame rate = "+frameRate+" per "+Time_unit+"\n"
 +"Frame interval = "+frameInterval+" "+Time_unit+"\n"
 +"Pixel size = "+pixelSize+" "+X_unit+"\n"
 +"Units of speed are "+X_unit+" per "+Time_unit+"\n"
 +"Total time = "+total_time+" "+Time_unit+"\n"
 +"RBC size in pixels = "+ RBC_size_in_pixels+"\n"
 +"LoG filter size in pixels = "+ radius_of_LoG_filter_in_pixels+"\n"
 +"Radius of mean filter in pixels used to measure mean RBC intensity = "+ radius_of_mean_filter_in_pixels
 );

*// Apply slicewise mean filter and slicewise LoG*
run("CLIJ2 Macro Extensions");
GPU_assisted_mean_by_slice(image_name, radius_of_mean_filter_in_pixels); *// mean filter each slice - this is used to determine the mean intensity of a candidate RBC*
GPU_assisted_LoG_by_slice(image_name, radius_of_LoG_filter_in_pixels);
run("Tile");

*// Alow the user to select the prominence used to detect maxima using the first slice*
*// NOTE: Maxima are ignored if they do not stand out from the surroundings by more than*
*// this value (called the "noise tolerance" in previous versions of the Find Maxima plugin)*
prominence = default_prominence_for_maxima_detection;
waitForUser("Make a note of the prominence value that you select in the next dialog");
selectWindow("slicewise_ LoG_"+image_name);
setSlice(1);
run("Find Maxima...", "prominence="+prominence+" strict exclude output=[Point Selection]"); *// creates a multipoint ROI*
run("Select None");
run("Find Maxima...");
prominence = getNumber("Noise prominence to use:", default_prominence_for_maxima_detection);

*// Compute the mean intensities of the candidate RBCs by simply looking up the mean filter value at the candidate locations*
run("Set Measurements...", "area mean redirect=None decimal=3");
selectWindow("slicewise_mean_filtered_"+image_name);
setSlice(1);
run("Restore Selection");
run("Measure");
run("Tile");

*// Find the mean of these mean intensities and use this as the default spot mean threshold*
spot_mean = Table.getColumn("Mean");
Array.getStatistics(spot_mean, min, max, mean, stdDev);
close("Results");

spot_mean_threshold = mean;

*// Now allow the user to try different values for the threshold*
**do** {
 selectWindow("slicewise_ LoG_"+image_name);
 setSlice(1);
 run("Find Maxima...", "prominence="+prominence+" strict exclude output=[Point Selection]"); *// creates a multipoint ROI*
 selectWindow("slicewise_mean_filtered_"+image_name);
 setSlice(1);
 run("Restore Selection");
 getSelectionCoordinates(x, y);

 *// delete all points from the multipoint ROI that do not satisfy the threshold*
 **for** (count=0; count < x.length; count++){
 **if**(getPixel(x[count], y[count]) < spot_mean_threshold){
 setKeyDown("alt"); *// delete this point*
 makePoint(x[count], y[count]);
 }
 }

 selectWindow(image_name);
 setSlice(1);
 run("Restore Selection");
 run("Properties... ", " stroke=yellow point=Circle size=Large"); *// display the candidate RBCs as yellow circles*
 waitForUser("Use Ctrl+Shift+A and Ctrl+Shift+E to toggle the overlay\nClick OK to continue");

 answer = getBoolean("Do you want to change the spot threshold?");
 **if** (answer == **true**) {
 spot_mean_threshold = getNumber("Enter threshold value", mean);
 }
}
**while** (answer == **true**);

*// Apply the selected prominence and threshold to all frames*
spot_detect(image_name, prominence, spot_mean_threshold);

close("slicewise_ LoG_"+image_name);
close("slicewise_mean_filtered_"+image_name);
run("Tile");


**function** GPU_assisted_LoG_by_slice(input_image, radius) {
 *// Compute the LoG*

*of each slice*
 Ext.CLIJ2_clear();
 image1 = input_image;
 Ext.CLIJ2_push(image1);
 image2 = "Gaussian_filtered"+input_image;
 sigma_x = radius;
 sigma_y = radius;
 sigma_z = 0;
 Ext.CLIJ2_gaussianBlur3D(image1, image2, sigma_x, sigma_y, sigma_z);
 Ext.CLIJ2_pull(image2);
 run("Laplace Filter"); *// This is a 2D Laplace filter applied to each slice*
 run("Enhance Contrast...", "saturated=0 normalize process_all");
 close("Gaussian_filtered"+input_image);
 run("Invert", "stack");
 rename("slicewise_ LoG_"+input_image);
}


**function** GPU_assisted_mean_by_slice(input_image, radius) {
 *// Mean filter each slice*
 Ext.CLIJ2_clear();
 image1 = input_image;
 Ext.CLIJ2_push(image1);
 image2 = "slicewise_mean_filtered_"+input_image;
 radius_x = radius;
 radius_y = radius;
 radius_z = 0.0;
 Ext.CLIJ2_mean3DBox(image1, image2, radius_x, radius_y, radius_z);
 Ext.CLIJ2_pull(image2);
}


**function** spot_detect(input_image, prominence, spot_mean_mean) {
 *// Apply the selected prominence and spot mean threshold to ALL slices*

 roiManager("reset");
 setBatchMode("hide");

 selectWindow(input_image);
 number_of_frames = nSlices;

 **for** (slice = 1; slice <= number_of_frames; slice++) {
 selectWindow("slicewise_ LoG_"+input_image);
 setSlice(slice);
 run("Find Maxima...", "prominence="+prominence+" strict exclude output=[Point Selection]"); *// creates a multipoint ROI*
 selectWindow("slicewise_mean_filtered_"+input_image);
 setSlice(slice);
 run("Restore Selection");
 getSelectionCoordinates(x, y);

 *// delete all points from the multipoint ROI that do not satisfy the threshold*
 **for** (i=0; i<x.length; i++){
 **if**(getPixel(x[i], y[i]) < spot_mean_threshold){
 setKeyDown("alt"); *// delete this point*
 makePoint(x[i], y[i]);
 }
 }
 roiManager("Add"); *// add this multipoint ROI to the ROI Manager*
 }


 *// Generate a binary stack with binary 1 indicating the location of each RBC*
 newImage("RBC_points_"+input_image, "8-bit black", getWidth(), getHeight(), number_of_frames);

 **for** (i = 0; i < roiManager("count"); i++) {
 selectWindow("RBC_points_"+input_image);
 roiManager('select', i); *// will automatically select the correct slice of "RBC masks"*
 run("Create Mask");
 run("Copy");
 close("Mask");
 selectWindow("RBC_points_"+input_image);
 run("Paste");
 }

 setBatchMode("exit and display");

 run("Divide...", "value=255.000 stack");
 run("Enhance Contrast", "saturated=0.35");

 *// Dilate each slice to create a blob for each RBC*
 run("Morphological Filters (3D)", "operation=Dilation element=Ball x-radius=1 y-radius=1 z-radius=0"); *// z-radius=0 ensures this happens slice by slice*
 rename("RBC_masks_"+input_image);
 run("Enhance Contrast", "saturated=0.35");
}

# Software S4

## vessel_diameter.ijm

*/**
 ** Author: Andrew Mehnert, Centre for Ophthalmology & Visual Science, The University of Western Australia*
 ***
 ** Synopsis: Measures the diameter of selected vessels in an image stack using the FWHM method*
 ** 1. The pixel size must be correctly set in the image metadata*
 ** 2. The user can select the channel and frames/slices to use*
 ** 3. The user draws a line ROI across the diameter of each vessel to be measured (the ROI set is automatically saved)*
 ** 4. The user-defined constant "line_ROI_width" defines the width of the line used to extract the intensity profile*
 ** 5. The script processes each ROI in turn*
 ** (i) A table is created for the ROI*
 ** (ii) The intensity profile values for each frame are added as columns to the table*
 ** (iii) The FWHM value is computed for each profile and added to the last row of the table*
 ** (iv) The pixel size is written to the last column*
 ** (iv) The table is saved to a CSV file*
 ***
 ** History: 11/10/2022 - V1.3*
 ***
 **/*

setOption("JFileChooser", **true**);
run("Close All");
roiManager("reset");
close("Results");
close("ROI Manager");

*// User-defined constants*
line_ROI_width = 10; *// Width of the line ROI used to extract the intensity profile for which the FWHM is calculated*


**function** replace_spaces_with_underscore(string) {
 fixed_string = replace(string, " ", "_");
 **return** fixed_string;
}


*// Ask user to select the nd2 file to process*
filepathname = File.openDialog("Select the RBC \"Aligned.nd2\" file to process");
File.getNameWithoutExtension(filepathname);
filename_without_extension = replace_spaces_with_underscore(File.getNameWithoutExtension(filepathname));
directory = File.directory;

*// Load as virtual stack*
run("Bio-Formats","open=[/"+filepathname+"] autoscale color_mode=Default rois_import=[ROI manager] view=Hyperstack stack_order=XYCZT use_virtual_stack");
image_name = filename_without_extension;
rename(image_name);

*// Load the user-specified channel and range of slices as a stack*
waitForUser("Info", "Please review the stack to decide which frames and channel you want to use");
run("Duplicate...");
run("Enhance Contrast...", "saturated=0.35 normalize process_all use"); *// linearly stretch intensities over entire bit range*
close(image_name);
rename(image_name);

*// Get pixel size and frame rate from the metadata and display an informational dialog box*
Stack.getUnits(X_unit, Y_unit, Z_unit, Time_unit, Value_unit);
getPixelSize(unit, pixelWidth, pixelHeight);
frameInterval = Stack.getFrameInterval(); *// in Time_unit*
frameRate = 1/frameInterval; *// in 1/Time_unit*
pixelSize = pixelWidth;
number_of_frames = nSlices;
total_time = number_of_frames * frameInterval;

showMessage("Properties for the stack", "Frame rate = "+frameRate+" per "+Time_unit+"\n"
 +"Frame interval = "+frameInterval+" "+Time_unit+"\n"
 +"Pixel size = "+pixelSize+" "+X_unit+"\n"
 +"Units of speed are "+X_unit+" per "+Time_unit+"\n"
 +"Total time = "+total_time+" "+Time_unit);

*// Perform a mean intensity z-projection and ask the user to add line ROIs tot ROI Manager*
run("Z Project...", "projection=[Average Intensity]");
setTool("line");
run("Tile");
run("Synchronize Windows");
waitForUser("Info", "Draw a line ROI across each vessel that you want to measure.\nAdd each ROI to the ROI Manager.\n \nAlternatively load in an existing ROiSet zip file.\n \nClick OK to continue");
roiManager("save", directory+filename_without_extension+"_diameter_RoiSet.zip")

*// Concatenate the mean intensity z-projection to the stack*
selectWindow(image_name);
run("Concatenate...", "open image1="+image_name+" image2=AVG_"+image_name+" image3=[-- None --]");
rename(image_name);

*// Extract line ROI profiles to tables along with the corresponding estimates of FWHM*
run("Select None");
number_of_frames = nSlices;
number_of_rois = roiManager('count');
roiManager("Deselect");
roiManager("Set Line Width", line_ROI_width);

setBatchMode("hide");
**for** (roi_number = 0; roi_number < number_of_rois; roi_number++){
 showProgress(roi_number, number_of_rois-1);
 selectWindow(image_name);
 roiManager('select', roi_number);
 run("Properties... ", " width="+line_ROI_width);
 roi_name = Roi.getName;
 Table.create("Table_for_"+roi_name);
 Plot.create("FWHM_for_"+roi_name, "Frame", "Width");

 frame_fwhm = newArray(number_of_frames);
 **for** (i = 1; i <= number_of_frames; i++) {
 selectWindow(image_name);
 roiManager('select', roi_number);
 setSlice(i);
 profile = getProfile();

 **if** (i < number_of_frames) {
 Table.setColumn("Frame "+i, profile, "Table_for_"+roi_name);
 }
 **else** {
 Table.setColumn("AVG Frame", profile, "Table_for_"+roi_name);
 }


 Array.getStatistics(profile, min, max, mean, stdDev);
 **for** (c=0; c<profile.length; c++) profile[c] = (profile[c] - min) / (max - min); *// normalise*
 fwhm = getFWHMFromProfiles(profile, profile.length, pixelSize);
 *//Array.print(fwhm);*
 frame_fwhm[i-1] = fwhm[0];

 **if** (i < number_of_frames) {
 Table.set("Frame "+

i, profile.length-1, fwhm[0], "Table_for_"+roi_name);
 }
 **else** {
 Table.set("AVG Frame", profile.length-1, fwhm[0], "Table_for_"+roi_name);
 }

 }
 Table.set("Pixel size", 0, pixelSize, "Table_for_"+roi_name);
 Table.update;

 Table.save(directory + filename_without_extension + "_ROI_" + (roi_number + 1) + "_" + roi_name + "_FWHM.csv");
 close("Table_for_"+roi_name);
 Plot.add("line", frame_fwhm);
 Plot.update();
}

setBatchMode("exit and display");

run("Images to Stack", " title=FWHM_for use");
rename("ROI_FWHM_for_"+image_name);
saveAs("tif", directory+"ROI_FWHM_for_"+image_name);


*/**
 ** Code below for FWHM is from the following paper:*
 ***
 ** McDowell, K., Berthiaume, A., Tieu, T., Hartmann, D., & Shih, A. (2020).*
 ** "VasoMetrics: Unbiased spatiotemporal analysis of microvascular diameter in multi-photon imaging applications".*
 ** Quantitative Imaging In Medicine And Surgery, 11(3), 969-982. doi:10.21037/qims-20-920*
 ***
 **/*


**function** getIntersection(centerX, centerY, radius, slope, yInt) {
 a = pow(slope, 2) + 1;
 b = 2 * ((slope * yInt) - (slope * centerY) - centerX);
 c = pow(centerY, 2) - pow(radius, 2) + pow(centerX, 2) - (2 * yInt * centerY) + pow(yInt, 2);
 x1 = (-1 * b + sqrt(pow(b, 2) - 4 * a * c)) / (2 * a);
 x2 = (-1 * b - sqrt(pow(b, 2) - 4 * a * c)) / (2 * a);
 **return** newArray(x1, x2);
}

**function** index(a, value) {
 **for** (i=0; i<a.length; i++) **if** (a[i]==value) **return** i;
 **return** -1;
}

**function** getProfilesForSlice(sliceNum) {
 profiles = newArray;
 **for** (i = 0; i < roiManager("count"); i++) {
 roiManager("select", i);
 *// Obtain the intensity profile and normalize it*
 getDimensions(width, height, channels, slices, frames);

 **if** (slices > 1) {
 setSlice(sliceNum);
 } **else** **if** (frames > 1) {
 Stack.setFrame(sliceNum);
 }

 profile = getProfile();
 Array.getStatistics(profile, min, max, mean, stdDev);
 **for** (c=0; c<profile.length; c++) profile[c] = (profile[c] - min) / (max - min);

 profiles = Array.concat(profiles, profile);
 }
 **return** profiles;
}

**function** getFWHMFromProfiles(profiles, profileLength, pixelScale) {
 fwhms = newArray;
 **for** (i = 0; i < (profiles.length / profileLength); i++) {
 profile = Array.slice(profiles, i * profileLength, (i+1) * profileLength - 1);
 *// Obtain the FWHM value for this profile*
 Array.getStatistics(profile, min, max, mean, stdDev);
 halfMax = max / 2;


 intersects = getYIntersects(halfMax, profile);
 Array.getStatistics(intersects, min, max, mean, stdDev);

 fwhm = (max - min) * pixelScale;

 *// Determine the derivative of this profile and use it to expand the bounds of FWHM height*
 derivative = newArray;
 **for** (x = 0; x < (profile.length - 1); x++) derivative[derivative.length] = profile[x+1] - profile[x];
 copiedProfile = Array.copy(profile);
 Array.sort(copiedProfile);
 median = (copiedProfile[floor((copiedProfile.length - 1) / 2)] + copiedProfile

[round((copiedProfile.length - 1) / 2)]) / 2;
 intersects = getYIntersects(0, derivative);
 leftIntChange = 0;
 rightIntChange = profile.length - 1;
 **for** (x = 0; x < intersects.length; x++) {
 **if** (intersects[x] > leftIntChange && min - intersects[x] > 0) leftIntChange = intersects[x];
 **if** (intersects[x] < rightIntChange && intersects[x] - max > 0) rightIntChange = intersects[x];
 }

 *// Using the adjusted intersects, recalculate the half max and find the x distance*
 Array.getStatistics(profile, min, max, mean, stdDev);
 halfMax = (max - minOf(profile[leftIntChange], profile[rightIntChange])) / 2;
 *//halfMax = max / 2;*
 fwhm = fwhmFromProfile(profile, halfMax, ((rightIntChange + leftIntChange) / 2), **false**) * pixelScale;
 fwhms[fwhms.length] = fwhm;
 }
 **return** fwhms;
}

**function** getCLineLength(x, y, imgHeight) {
 **if** (getBoolean("Select cross-line length method", "Automatically Calculate Length", "Manually Enter Length")) {
 getDimensions(width, height, channels, slices, frames);
 **if** (slices > 1 || frames > 1) {
 run("Z Project...", "projection=[Max Intensity] all");
 } **else** {
 run("Duplicate...", " ");
 }

 run("Median...", "radius=10 stack");
 maxPeakDist = 0;
 **for** (i = 0; i < x.length - 1; i++) {
 invM = -1 / ((y[i+1] - y[i]) / (x[i+1] - x[i]));
 invB = ((y[i+1] + y[i])/2) + (-1 * invM * ((x[i+1] + x[i]) / 2));

 **if** (invM > 0) {
 makeLine(0, invB, (imgHeight - invB) / invM, imgHeight);
 } **else** {
 makeLine(0, invB, (0 - invB) / invM, 0);
 }

 profile = getProfile();
 dist = sqrt(pow(((x[i+1] + x[i]) / 2) - 0, 2) + pow(((y[i+1] + y[i])/2) - invB, 2));
 sample = Array.slice(profile,dist-10,dist+10);
 Array.getStatistics(sample, min, max, mean, stdDev);
 halfMax = max / 2;
 fwhm = fwhmFromProfile(profile, halfMax, dist, **true**);

 peakDist = fwhm + (0.65 * fwhm);
 **if** (peakDist > maxPeakDist) maxPeakDist = peakDist;
 }
 close();

 **if** (maxPeakDist == 0 || maxPeakDist > 80) {
 **return** getNumber("Automatic Line Length Calculation Failed. Please input length for crosslines (in pixels).", 20) / 2;
 } **else** {
 **return** maxPeakDist / 2;
 }
 } **else** {
 **return** getNumber("Please input length for crosslines (in pixels).", 20) / 2
 }
}


**function** fwhmFromProfile(profile, targetY, vesselCenterX, minDist) {
 intersects = getYIntersects(targetY, profile);
 **if** (targetY < 0.2) **return** **NaN**;
 **if** (intersects.length < 2) **return** **NaN**;

 leftX = intersects[0];
 rightX = intersects[intersects.length - 1];
 **for** (

x = 0; x < intersects.length; x++) {
 **if** (minDist && intersects[x] < vesselCenterX && vesselCenterX - intersects[x] < vesselCenterX - leftX) leftX = intersects[x];
 **if** (minDist && intersects[x] > vesselCenterX && intersects[x] - vesselCenterX < rightX - vesselCenterX) rightX = intersects[x];
 **if** (!minDist && intersects[x] < vesselCenterX && vesselCenterX - intersects[x] > vesselCenterX - leftX) leftX = intersects[x];
 **if** (!minDist && intersects[x] > vesselCenterX && intersects[x] - vesselCenterX > rightX - vesselCenterX) rightX = intersects[x];
 }

 **return** rightX - leftX;
}

**function** getYIntersects(targetY, fx) {
 intersects = newArray;
 **for** (c=0; c < (fx.length - 1); c++) {
 profileSlope = (fx[c+1] - fx[c]);
 profileYInt = fx[c] + (-1 * profileSlope * c);
 xInt = (targetY - profileYInt) / profileSlope;
 **if** (xInt >= c && xInt <= (c+1)) {
 intersects[intersects.length] = xInt;
 }
 }
 **return** intersects;
}
